# Supplementary material for: Patterns of Intron Gain and Loss in Fungi
Source: PLoS Biol. 2004 Nov 30;2(12):e422. doi: 10.1371/journal.pbio.0020422 (PMC532390; doi:10.1371/journal.pbio.0020422)
Supplement: Table S1 — Also available at http://genes.mit.edu/NielsenEtAl/. (4.3 MB ZIP). [file pbio.0020422.st001.zip › NielsenEtAl/html/567.html]

AN0423.1.NCU08384.1.MG03648.1.FG01523.1


```
 CLUSTAL W (1.82) Multiple Sequence Alignments - Introns Inserted


Sequence 1: NCU08384.1	322 aa
Sequence 2: MG03648.1	324 aa
Sequence 3: AN0423.1	319 aa
Sequence 4: FG01523.1	324 aa
Alignment Length: 325 aa
Number Identitical Residues: 160 aa
Alignment Score (without introns) 8508


MG03648.1 	-MASPTMKLNNGLDMPQVGFGLWKVENSVCADVVYNAIKAGYRLFDGACD1YGNEVECGQ
NCU08384.1	--MVPAIKLNSGFDMPQVGFGLWKVDGSIASDVVYNAIKAGYRLFDGACD1YGNEVECGQ
FG01523.1 	MVNVPNIKLNSGFDMPQIGFGLWKVD-DNCAEVVYNAIKAGYRLLDGACD1YGNEKACGE
AN0423.1  	-MSPPTVKLNSGYDMPLVGFGLWKVNNDTCADQVYEAIKAGYRLFDGACD1YGNEVEAGQ
          	    * :***.* *** :*******:.. .:: **:********:***** ****  .*:

MG03648.1 	GVKRAIDEGLVKREELFIVSKLWNTFHDGERVEPIVKKQLADWGIEYFDLYLIHFPVALE
NCU08384.1	GVARAIKEGIVKREELFIVSKLWNTFHDGDRVEPIVRKQLADWGLEYFDLYLIHFPVALE
FG01523.1 	GVARAIKEGIVKREDLFIVSKLWQTYHDKKNVEPITRRQLADWQIDYFDLFLIHFPVALE
AN0423.1  	GVARAIKEGIVKRSDLFIVSKLWNSFHDGERVEPIARKQLSDWGIDYFDLYIVHFPVSLK
          	** ***.**:***.:********:::** ..****.::**:** ::****:::****:*:

MG03648.1 	YVDPSVRYPPGWHYDDAGTEIRPSKASIQETWTAMEKLVDAGLSKAIGVSNFQAQLLYDM
NCU08384.1	YVDPSVRYPPGWHFDGK-SEIRPSKATIQETWTAMESLVEKGLSKSIGVSNFQAQLLYDL
FG01523.1 	YVDPEVRYPPGWHYDDAGTEIRWSKATNQETWEGMEDLVEKGLAKSIGISNFQAQGIYDM
AN0423.1  	YVDPEVRYPPGWENAEG--KVELGKATIQETWTAMESLVDKGLARSIGISNFSAQLLLDL
          	****.*******.      ::. .**: **** .**.**: **:::**:***.** : *:

MG03648.1 	LRYARIRPATLQIEHHPYLVQQRLIEACKTEGIVVTAYSSFGPASFKEFNMEHAEALTPL
NCU08384.1	LRYAKVRPATLQIEHHPYLVQQNLLNLAKAEGIAVTAYSSFGPASFREFNMEHAQKLQPL
FG01523.1 	LKYAKIRPATLQVELHPYHQQSELVSLAKAEGIALTAYSSFGPTGFIELDMDIAKSASPL
AN0423.1  	LRYARIRPATLQIEHHPYLTQERLVTFAQREGIAVTAYSSFGPLSFLELSVKQAEGAPPL
          	*:**::******:* ***  *..*:  .: ***.:******** .* *:.:. *:   **

MG03648.1 	LEEPTIVKLAEKYGKDPGQVLLRWATQRGLAVIPKSSREKTMKSNFEAVGWDMEDSDIKT
NCU08384.1	LEDPTIKAIGDKYNKDPAQVLLRWATQRGLAIIPKSSREATMKSNLNSLDFDLSEEDIKT
FG01523.1 	MQHEVFTTLASKYNKTSAQVLLRWATQQGLAVIPKSTTPQYMAQNFDCFEWSIDEEDMKR
AN0423.1  	FEHPVIKDIAEKHGKTPAQVLLRWATQRGIAVIPKSNNPARLLQNLDVVGFDLEDGELKA
          	::. .:  :..*:.* ..*********:*:*:****.    : .*:: . :.:.: ::* 

MG03648.1 	ISALDKGIRFNQPAN0YFSTDKLWIFG
NCU08384.1	ISGFDRGIRFNQPTN0YFSAENLWIFG
FG01523.1 	ISKMNLNLKFNKPTN0YFPTEKLWIFG
AN0423.1  	ISDLDKGLRFNDPPN0YGLPITIF---
          	** :: .::**.*.* *  . .::
```
